# Continuous Authentication of Automotive Vehicles Using Inertial Measurement Units

**DOI:** 10.3390/s19235283

**Published:** 2019-11-30

**Authors:** Gianmarco Baldini, Filip Geib, Raimondo Giuliani

**Affiliations:** 1 European Commission, Joint Research Centre, 21027 Ispra, Italy; filip9geib@gmail.com (F.G.); raimondo.giuliani@ec.europa.eu (R.G.); 2 Faculty of Electrical Engineering, Czech Technical University in Prague, 160 00 Praha, Czech Republic

**Keywords:** security, authentication, Inertial Measurement Units, road transportation

## Abstract

The concept of Continuous Authentication is to authenticate an entity on the basis of a digital output generated in a continuous way by the entity itself. This concept has recently been applied in the literature for the continuous authentication of persons on the basis of intrinsic features extracted from the analysis of the digital output generated by wearable sensors worn by the subjects during their daily routine. This paper investigates the application of this concept to the continuous authentication of automotive vehicles, which is a novel concept in the literature and which could be used where conventional solutions based on cryptographic means could not be used. In this case, the Continuous Authentication concept is implemented using the digital output from Inertial Measurement Units (IMUs) mounted on the vehicle, while it is driving on a specific road path. Different analytical approaches based on the extraction of statistical features from the time domain representation or the use of frequency domain coefficients are compared and the results are presented for various conditions and road segments. The results show that it is possible to authenticate vehicles from the Inertial Measurement Unit (IMU) recordings with great accuracy for different road segments.

## 1. Introduction

Authentication is an important function in security as it ensures that the identity of an entity is recognized by a system whose services the entity would like to access. Authentication is usually combined with other security functions like authorization where the entity, once authenticated, is authorized to access specific services or data. The verification of the identity can be implemented using different means including information known by the entity (e.g., login/password) based on what the entity is (e.g., biometric features). This information can be provided only once at the beginning of the interaction between the entity and the system or more than once either periodically or in a continuous way. The latter methods are considered more robust than one time login against external attacks because an attacker, who aims to impersonate the real entity, has to provide authentication information more than once, which is considerably more difficult [1,2]. On the other side, the request for an additional login/password after the initial login may negatively impact the usability of the human-machine interface [3]. In this context, the concept of continuous authentication has been mostly proposed in the literature for human authentication. It makes use of the physiological and behavioral biometrics using built-in sensors and accessories. For example, the research results in the literature have proven that persons can be uniquely identified by the way they use mobile devices [1,4], they type on a keyboard [5] or perform different activities. Details on the literature results are provided in the following paragraphs.

Continuous authentication can be performed in the application scenarios where the entity must access services to the system for a considerable amount of time and where the biometrics information can be collected from the authentication system. For example, smart city application scenarios could benefit from this approach [6]. Various techniques to implement continuous authentication for persons have been proposed and validated by the research community in recent times. While continuous authentication has been researched for human beings in recent years, there has been limited attention to other entities especially in the transportation domain (e.g., a study on the identification of transport modes rather than transport vehicles is Reference [7]). Details on the existing related work are presented in Section 2.

### Our Contribution

This paper proposes a proof of concept on the application of the continuous authentication technique (used in the literature to identify human beings) to the road transportation domain and more specifically to the problem of device identification where the device is actually a vehicle (i.e., a car in this paper). This paper investigates the possibility of authenticating a car on the basis of its “biometrics” derived from the way the car responds to the irregularities (e.g., potholes, bumpers) of the road surface. The rationale is that the components (e.g., bumpers) of different cars provide a different response to the same segment of the road surface. Such responses can be evaluated by using the digital output provided by Inertial Measurement Units (IMU) and its components (e.g., accelerometers, gyroscope) installed in a car in a similar way that continuous authentication is implemented for human beings using wearable sensor or smartphones (which also contain IMUs). This paper presents the evaluation results for the application of techniques derived from the literature on continuous authentication. In particular, two different approaches are compared both from an identification accuracy and an execution time point of view. The results presented in this paper show that each vehicle can be uniquely identified with high accuracy by the analysis of the data collected by Inertial Measurement Unit (IMU) accelerometers and gyroscopes while the car is driving. To the knowledge of the authors, it is the first time that the concept of continuous authentication is applied to vehicles (i.e., car).

A potential scenario for the use of continuous authentication of vehicles (or cars as the two terms will be used with the same meaning in the rest of this paper) is based on Intelligent Transport Systems (ITS) applications, where the authentication of the vehicle is needed a number of times during the vehicle trip like “insurance as you drive” or electronic tolling. The continuous authentication can complement (as multi-factor) authentication based on cryptographic means or replace it when the set-up of a Public Key Infrastructure (PKI) is too costly or complex to achieve. Then, the motivation of the paper is to propose a different form of device (i.e., the car) identification and authentication when a cryptographic mean cannot be applied or in case of the compromise of the cryptographic system (e.g., PKI) and the presence of an invalid cryptographic information (e.g., certificate) in the vehicle. In other words, this approach can be applied in all the cases where a cryptographic mean is not available, either because it is not implemented or because it has been compromised. The authors agree that, in the majority of the cases, a cryptographic solution is the primary solution because of its proven effectiveness. In addition, it can be noted that there are also examples of regulations for commercial vehicles were data is manipulated and tampered even if the cryptography authentication is not compromised [8]. Because the form of authentication described in this paper is based on the data analysis, it can mitigate such issues. The proposed approach is based on the consideration that modern vehicles are increasingly equipped with a variety of sensors including IMU, which are used for a variety of purposes but mostly to improve transportation comfort and driveability [9]. Many authors have also proposed the use of smartphones mounted in the vehicle for easier accessibility to the data generated by the built-in IMU [10].

The deployment scenario is similar to the continuous authentication of a human-using behavioral metric. In an initial training phase, data samples are acquired through remote communication by an entity external (e.g., a cloud application) to the vehicle. Then, discriminating features are identified and extracted from the data. In a subsequent phase, an authentication system (either based on the same cloud application or connected to it) uses a smaller data subset and compares it against the training model for identification. The two key performance metrics are (a) the identification accuracy and (b) the processing time for authentication, which is proportional to the amount of the data, which must be processed. Because IMU data ca have hundreds of samples per second, a dimensionality reduction is necessary, while preserving the identification accuracy. In this paper, we used professional IMUs to record the data (while driving) originating from accelerometers and gyroscopes with high sample rates. In the subsequent analysis, the sampling rate is decreased to the rate commonly used by mass market smartphones.

In this initial study, the focus is to investigate the capability of authenticating the vehicle (i.e., car) in similar conditions and on the same road surface. In a practical application of this concept, different variables can influence the classification accuracy including the driver, different loads in vehicle (number of passengers), aging of the vehicle (e.g., tyre consumption) and different environmental conditions (e.g., rain, sunny weather). Each of these variables can influence the application of the method, but it is important to investigate the impact of each variable independently starting from a baseline. We also note that there is already an extensive literature on the identification of the driver and his/her driving style based on data collected by accelerometers (see References [7,11]) and it was not the intention to repeat such specific analysis here. Then, the focus of this paper was to establish a baseline where the most effective continuous authentication methods could be identified in a quantitative way. Future developments will evaluate the impact of each of the variables (see also Conclusions section).

The structure of this paper is as follows: Section 2 provides an overview of the related work on continuous authentication in all domains, authentication of vehicles and on the use of IMU to collect behavioral statistics in ITS. Section 3 describes the materials used in the experimental work. Section 4 describes the adopted methodology to collect the data and to perform the authentication. Section 5 provides the results of the implementation of the authentication where the impact of different hyper-parameters was quantitatively evaluated. Section 6 summarizes and discusses the key findings from the results and identify potential future developments.

## 2. Related Work

A very recent (2019) survey [12] reviews the techniques for continuous authentication using Internet of Things devices. Another review provides an overview of the state of art on continuous authentication using mobile devices [1]. Both surveys are specific to the problem of continuous authentication of human beings on the basis of behavioral bio-metrics rather than explicit and single phase authentication mechanisms like the password, a Personal Identification Number (PIN), or a secret pattern on the display of the smartphone. Both reviews highlight the weaknesses of the traditional approaches and the advantages of the continuous authentication approach. For example, passwords or PINs can be forgotten or systems may be vulnerable immediately after the initial login. In addition, some authentication mechanisms require the implementation of sophisticated Public Key Infrastructure (PKI)s, which are also vulnerable to attacks. To overcome these issues, the biometrics and security research communities have developed techniques for continuous authentication on mobile devices [1]. Most of these methods make use of physiological and behavioral biometrics, using built-in sensors and accessories such as the gyroscope, touch screen, accelerometer, orientation sensor and pressure sensor, to monitor and authenticate the identity of the user.

In this review of the state-of-the-art, we focus specifically on continuous authentication based on accelerometers and gyroscopes as these components are also used in this paper for vehicle authentication. This type of continuous authentication is based on the concept of identifying the gait of a human being while s(he) is performing a specific activity like walking, running or lifting an object. The data needed to perform the continuous authentication are often measured using the built-in components of a smartphone like accelerometers. Once the raw data are collected and measured, discriminative features are extracted, which are then fed into a classifier to distinguish users. This is a similar approach used in this paper. Various approaches are used in the literature and it is quite common to use either (or both) time domain and frequency domain representations of the recordings of the accelerometers and gyroscopes data. For example, the authors in Reference [13] extract statistical features from sensor readings, which are then used for user classification using a Support Vector Machine (SVM) machine learning algorithm. The statistical features used in [13] are mean, standard deviation, kurtosis, skewness, entropy and so on, both in the time domain and in the frequency domain. Then, from the methodology point of view, the approach used in Reference [13] is very similar to this paper with the fundamental difference that this paper focuses on vehicle authentication, which is a novel approach in the literature. In a similar way, the authors in Reference [14] have used the accelerometer data from a smartphone attached to a pocket of a person while walking. Each step was the gait, which could be used for authentication. An important element in the methodology was the segmentation of the accelerometer recordings to identify the repeated steps. Then, the identified segments were fed to a SVM classifier. The issue that persons are walking at different speeds was mitigated using Dynamic Time Warping (DTW). The appropriate segmentation of the recordings and the different speeds of the entity is also a problem addressed in this paper. A similar approach was also used by the authors in Reference [15], where the main coefficients of the frequency domain transform (based on FFT) of accelerometers data of walking persons were used for person authentication. Instead of using the Fast Fourier Transform (FFT), the authors have applied the Wavelet transform in Reference [16] in the context of a similar scenario of the previous papers: person authentications through mobile phone built-in accelerometers. Another recent paper, which presents continuous authentication of human beings using accelerometers recordings is Reference [17] which focuses on passive and continuous user authentication by analyzing and recognizing the unique characteristics of the physical activity patterns of human beings. A number of statistical features were extracted from the sensors recording—mean, standard deviation, skewness, kurtosis, energy and entropy in the time domain and frequency domain. Then, three machine learning algorithms (e.g., SVM, Random Forests and Decision Trees) were used for classification and authentication. As we have described in this section, there is an extensive literature on continuous authentication of human beings using accelerometers and gyroscopes and extraction of statistical features. The novel approach presented in this paper is to apply the techniques described above for the continuous authentication of vehicles which has never been investigated in the literature.

Authentication of vehicles is mostly based on cryptography means [18] and it has been applied to various interactions between vehicles and specific applications like the vehicle to the electric grid infrastructures in Reference [19] or for C-ITS applications [20]. In many cases, the authentication and authorization process is standardized as in the case of C-ITS domain [21,22,23]. Various cryptographic architectures are proposed for vehicular networks including PKI or Identity-based cryptography.

The use of IMU and their digital output in ITS applications has received considerable attention in recent years thanks to the increasing computing power of smartphones and their built-in IMU. On the other side, no papers have used such data to implement the continuous authentication of vehicles. The focus has been mostly on the estimation of the driver style (e.g., aggressive) using data from accelerometers in Reference [24] or (in a similar way) the driving behavior as in Reference [25]. A paper that is mostly similar to ours is Reference [7] where the transport mode (e.g., vehicle, bicycle) is identified on the basis of the digital output from IMUs, while this paper focuses on the identification of the specific vehicle in a specific transport mode.

Then, we can conclude that the idea of using the concepts of continuous authentication of human beings to vehicle is rather novel and it will be comprehensively explored in the rest of this paper.

## 3. Materials

**Figure 1:**
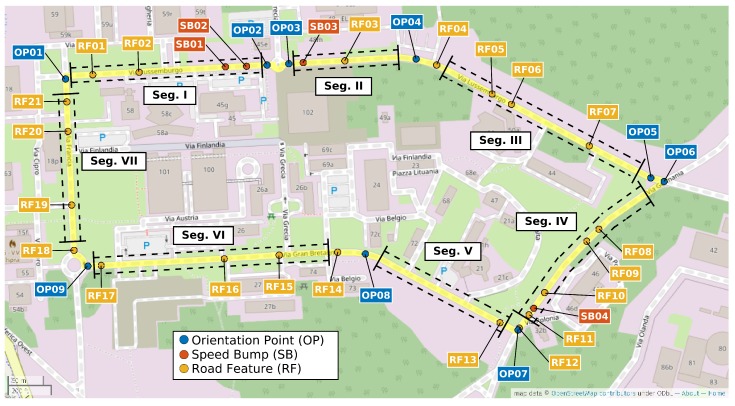
Map of the Experimental Lap (EL) with marked points and the most important segments.

**Figure 2:**
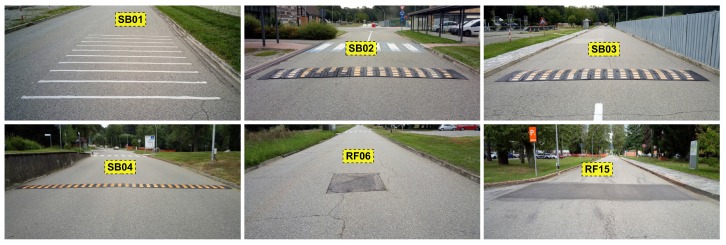
Photo documentation of the most significant points detected on the EL.

The goal of the experiment was to obtain cars’ driving characteristics under comparable driving conditions. We planned an Experimental Lap (EL) (in the rest of this paper, the term EL and lap will be used with the same meaning) inside the Joint Research Centre (JRC) area with conventional road rules and realistic traffic. This lap had a length of 2065 m and average driving time of 182.5 s (see Figure 1). To collect the measurements, every car had to drive this lap for at least twenty times in a row with just a tiny stop between each other (from 2 to 10 s). We distinguish and label the three types of points on the EL as follows—Orientation Point (OP) (e.g., any kind of left or right turn that we later used to separate the smaller parts of the EL), Speed Bump (SB) (e.g., four big speed bumps spread through the EL) and Road Feature (RF) (any roughness on the surface of the road, such as speed bump or an asphalt joint). Examples of those points with their descriptions and Global Navigation Satellite Systems (GNSS) positions are in Table 1. The disposition of the points is also marked in the map of the EL (see Figure 1), the photo documentation of the most significant ones is shown in Figure 2 and a description of the most significant points with the related latitude and longitude is provided in Table 1. The EL starts at OP01 and continues in clockwise.

**Figure 3:**
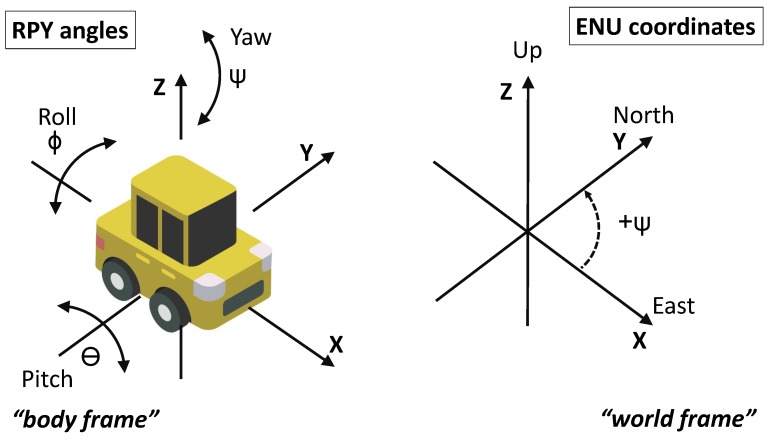
Alignment and orientation of IMU used during measurements.

**Figure 4:**
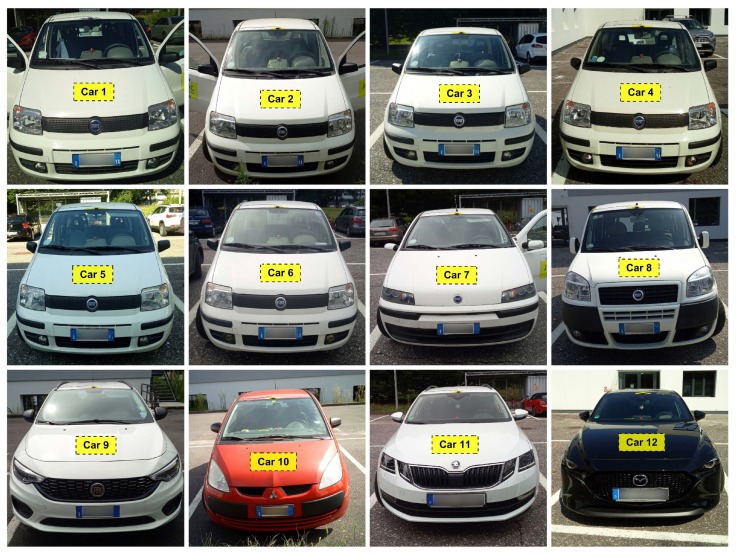
Photo documentation of the cars set used in this paper.

For the IMU, we used the microelectromechanical system based motion tracker supplied by Xsens with the model number MTi-100-2A8G4 (Xsens Technologies B.V., Enschede, The Netherlands). This sensor device is designed to measure the three axis acceleration and rate of turn in 2000 Hz sample rate and three axis magnetic field in 100 Hz sample rate. The IMU was mounted using a strong double sided foam tape at the same spot and orientation for every car. For details see Figure 3. We decided to place the sensor on the top of car’s dashboard in the middle of the car. No measuring tools were used and the alignment was fully empirical. The x-axis of the IMU was always pointing towards the driving direction and z-axis in the vertical direction. The car position was also recorded using the GNSS receiver with the antenna mounted on the car’s roof. The set used for this experiment consisted of twelve different cars. The specifications of the vehicles are listed in Table 2 and the photo documentation of those cars is in Figure 4. The same driver and co-driver were present in every car during the experimental data collection (i.e., driving the car) to minimise the potential bias of the driver. We highlight that half of the used vehicles were of the same model (Panda Active). The choice of such datasets with a predominance of vehicles of the same model and brand was intentional. While, this aspect is not quite relevant in the continuous authentication of human beings (because each human being is unique), this aspect is quite relevant in device authentication as discussed in the related literature [26], where it is discussed how intra-model classification is more difficult than inter-model classification (because components of the same model are the same or similar). To clarify—intra-model classification is when the devices are of the same model and brand (e.g., mobile phones of brand/model Sony Experia or Fiat Panda as in this scenario), while inter-model classification is when devices are of a different brand and/or models. We wanted to target the most difficult problem of intra-model classification rather than inter-model. This is why we have used a majority of vehicles of the same brand and mostly of the same model.

## 4. Methodology

### 4.1. Workflow

**Figure 5:**
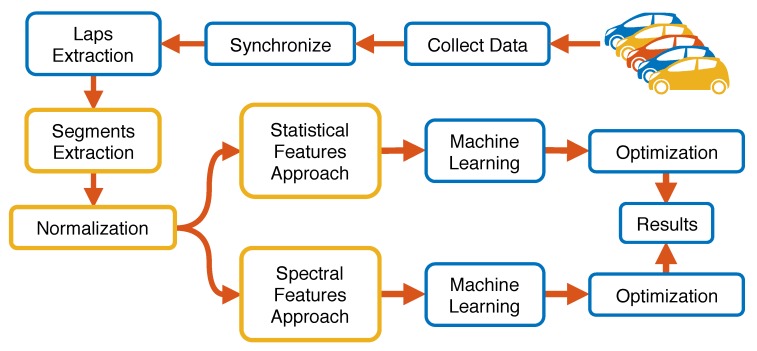
Methodology workflow.

A pictorial description of the methodology workflow is provided in Figure 5, where each step is described in detail in the following subsections.

### 4.2. Data Collection

The first step is to collect the data from each vehicle. As described in the Section 3, the same driver was used for all the recordings. The driving was performed in different environmental conditions (e.g., sunny days, rainy days) always on the same loop for 20 times. The data were recorded from the Xsens IMU with a sample rate of 2000 Hz, which is much higher than the sample rate available in commercial smartphones. For this reason, the sample rate was decimated to a lower sample rate as described in the Section 5. The car was driven at different speeds in the EL because of the varying traffic conditions.

### 4.3. Synchronization and Laps Extraction

**Figure 6:**
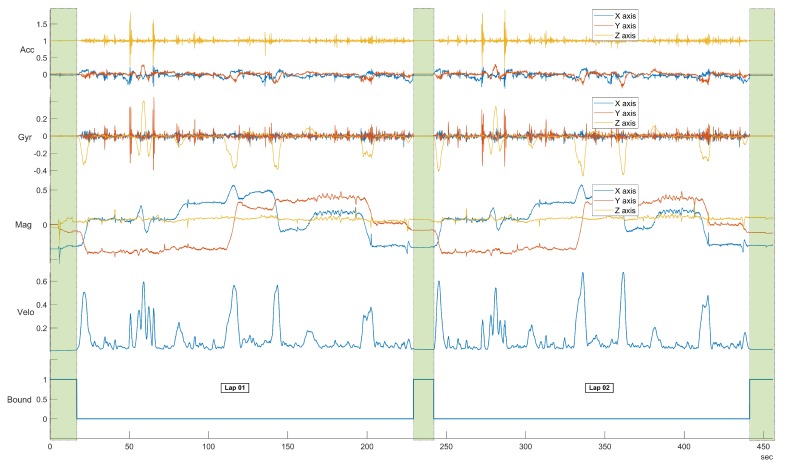
Detailed representation of the first two laps after synchronization. This plot shows that the proposed synchronization and laps extraction approach does not lose data between the laps (area highlighted with a green colour).

The second step is to synchronize the data recordings and to extract the identified laps. To facilitate this step, each lap driving was performed in the following way—the car was stationary on OP01 at the beginning of each lap with a running engine for 10 s. This initial Background Noise Window (BNW) segment was used for laps synchronization and extraction. To compute an overall motion of the car the Kalman filter is used to estimate three dimensional angular velocity from the gyroscope sampled to 20 Hz. The variance computed from the BNW is used as a filter parameter for the noise reduction. Then the computed angular velocity is normalized to only one dimension and the one-dimensional convolution is applied. The convolution matrix is normalized to 20 elements long (due to the 20 Hz down-sample frequency). The start and end of each lap is calculated using the value of the angular velocity, when it is less than the threshold for the specific time defined above. The output of this procedure is a time table with starting and ending times of each lap. A description of the pseudo algorithm is shown in Algorithm 1. The visualization of the synchronization and laps extraction for the first two laps is shown in Figure 6.
**Algorithm 1:** Detect laps and compensate different alignment for one car.
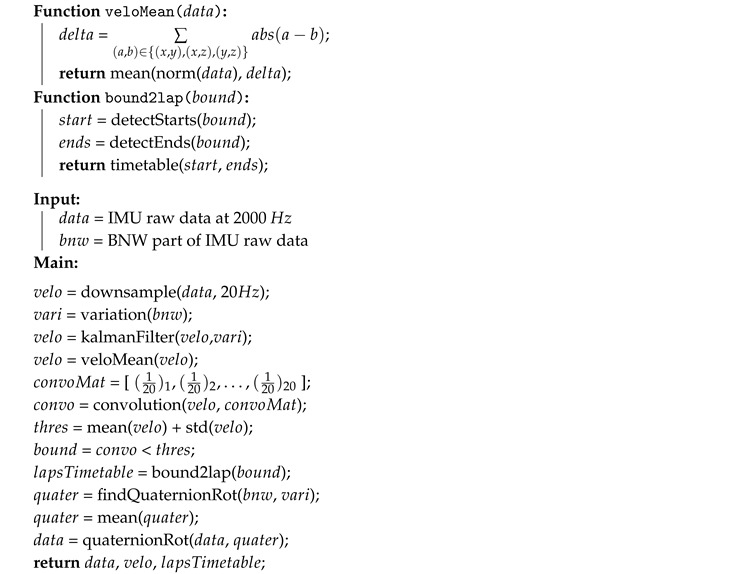


### 4.4. Segments Extraction

In the following step, the different road segments are extracted from each lap. The goal is to divide the entire lap in specific segments on which the classification process for vehicle continuous authentication is performed. The issue is already known in literature for continuous authentication of persons [13,14] and it is obviously based on the consideration that the speed of the car will never be exactly the same across laps in a similar way to a person walking or moving at different speeds. A possible solution to the different cars’ speed problem across laps is to re-sample the laps records to have the same number of data points. The result of such a squeezing effect is similar to the case in which every car has the same speed pattern in every lap. It is important to divide the lap into many smaller parts similar for every car and to squeeze them separately. For detecting those parts we took advantage of the shape of the EL with many curves. Every one of them and also the readings between them are considered as a separate parts.

**Figure 7:**
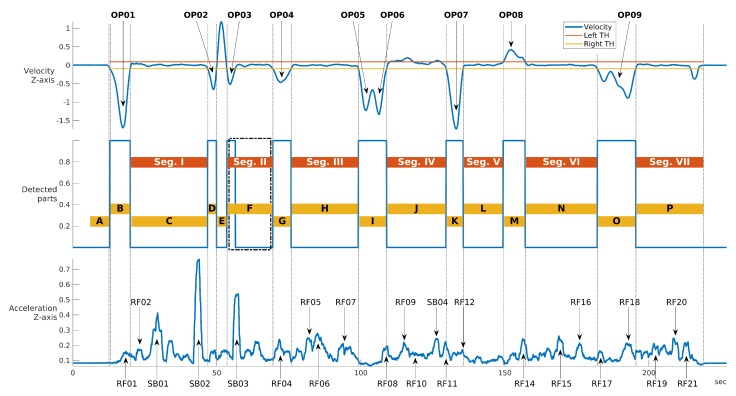
An example of the segments identified in a lap of the first car. The first plot shows the convoluted z-axis of the axial velocity with its positive (right turn) and negative (left turn) threshold. The Operational Point (OP) positions from Figure 1 are marked above in form of text arrows. The detected turns are shown at the second subplot (1 means turn and 0 means straight drive). The vertical gray lines separates different segments which are individually marked by yellow rectangles with alphabetical letters. Those letters represents segment names and stands for: A-Start, B-StartTurn, C-FastFirstBump, D-PreRound, E-RoundOne, F-SecodBump, G-RightCurve, H-WindowOne, I-CrossOne, J-VisitBump, K-CrossTwo, L-WindowTwo, M-LeftCurve, N-WindowThree, O-RoundTwo, P-WindowFour. The orange rectangles identify the seven segments used for the machine learning classification. The last subplot shows the z-axis of the acceleration with the identified EL road landmarks from the Figure.

To detect turns, the z-axis of angular velocity is used. It is negative during the right turn, positive during the left turn and very close to zero when the car drives straight. One dimensional convolution is applied. The turns are detected separately in every lap of current car to minimize the potential speed biases between those laps. The threshold is set on the sum of median and one fourth of standard deviation of the velocity in the current lap. Every velocity value between the negative threshold and the positive threshold is considered as a turn. This method also detects many smaller turns which are invalid for the next usage. The second level of detection is applied to erase those small turns. For every turn, the area between its velocity and threshold is computed. If this area is smaller than the length of this threshold, it is marked as a false turn and erased. The output of this procedure is a time table with the starting times of each detected segment for each lap. We were able to detect sixteen of them. For more information, see Figure 7. For the description of the pseudo algorithm, see Algorithm 2.

**Algorithm 2:** Detect laps parts for one car.

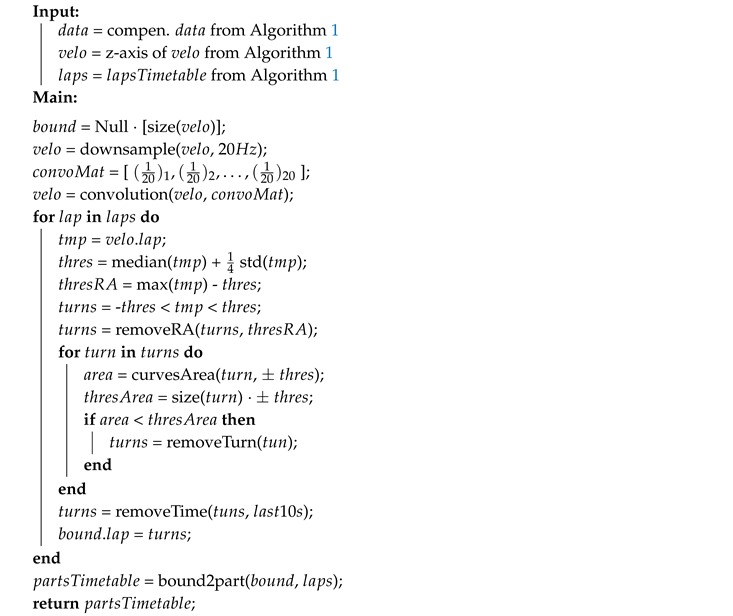



### 4.5. Segments Records Re-Sampling

For every segment, its longest occurrence is found between records of every car and its every lap. This maximal segment is used as a reference and re-sampled directly to the desired frequency. Then the algorithm executes throughout the rest of the segment occurrences (for all cars and laps) and re-sample them with individually computed sample rates so that it will match the length of the already re-sampled reference segment. Because the longest segment was chosen as the reference, the computed sample rate are always higher than the desired sample rate. For the description of the pseudo algorithm, see Algorithm 3. The sample rate of our measurements (i.e., 2000 Hz) determined the lower and upper frequency boundaries of re-sampling approach. We found that frequencies below 50 Hz were not enough to hold usable data quality for classification. On the contrary, frequencies above 500 Hz were too high for down-sampling only and required up-sampling for some particular segments. In addition, we note that a sample rate above 500 Hz is unrealistic for most of the mobile phones available in the market.

**Algorithm 3:** Squeeze separately data parts of all cars to lowest desired frequency.

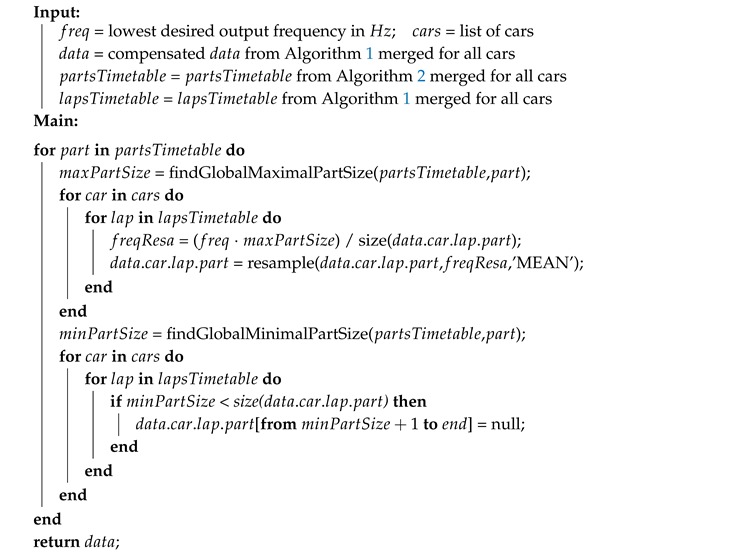



### 4.6. Description of the Classification Techniques

After the alignment and synchronization, the classification of the vehicles is performed using two different techniques. The first technique is based on the extraction of statistical features and it is presented in Section 4.6.1. The second technique is based on the application of the FFT transform and the analysis is conducted in the spectral domain representation and it is described in Section 4.6.2.

#### 4.6.1. Statistical Features Approach

To perform dimensionality reduction, statistical features are applied to each segment. Such statistical features are commonly applied to time series classification and in particular for continuous authentication as described in the related work in References [13,17]. The list of applied statistical features is shown in Table 3.

A brief description of the statistical features is provided here. Variance, Skewness and Kurtosis are moments of different levels of a specific quantitative measure of the shape of a function. Variance (second central moment) is the expectation of the squared deviation of a random variable from its mean. Skewness (third moment) is a measure of the asymmetry of the probability distribution of a real-valued random variable about its mean. Kurtosis (fourth moment) is a measure of the tailedness of the probability distribution of a real-valued random variable. The entropy state function is simply the amount of information (in the Shannon sense) that would be needed to specify the full microstate of the system and it can be used as a statistical measure of randomness. The Wavelet Spectral Shannon Entropy is the Shannon entropy calculated in the spectral representation of the IMU recording transformed using the wavelet transform. The Wavelet Spectral Log Entropy is the entropy calculated using a logarithmic scale on the spectral representation of the IMU recording transformed using the wavelet transform. The Permutation Entropy (PE) was initially proposed in Reference [27] and it has been applied in many domains from healthcare, to analyze EEG recordings [28], to the detection of mechanical faults [29]. PE [27] is a complexity measure for a time series *x* of *T* elements and an embedding dimension D≥2. The time series is embedded to a D-dimensional space $X_{t}=\left\{x\left(t\right),x\left(t+1\right),\ldots ,x\left(t+D-1\right)\right\}$, with *t* ranging from 1 to T−D+1. Given an ordinal set $R_{D}=\left\{r1,r2,\ldots ,rD\right\}$, where r1<r2<…<rD, there are D! permutations $\pi_{i}$, with *i* ranging from 1 to D!. Then, $X_{t}$ is mapped to $\pi_{i}$, so that {x(t),x(t+1),…,x(t+D−1)}↦{r1,r2,…,rD} and {x(t)≤x(t+1)≤…≤x(t+D−1)}. Probabilities $p_{i}$ of each $\pi_{i}$ are calculated as the number of occurrences of each $\pi_{i}$ out of the size of the dimensional space T−D+1. The permutation entropy of order D is then calculated with the formula $PE=-\sum_{i=1}^{D!}p_{i}\times \log p_{i}$, while the normalised permutation entropy is NPE=PE/log(D!). As the embedding dimension D≥2 is an important factor, two values of it has been used in our evaluation. Higher values are not considered because of the limitation on the size of the segments, which are imposed by the equations described above. Lower values are also not significant. Approximate entropy (ApEn) is a recently developed statistic quantifying regularity and complexity, which appears to have potential application to a wide variety of relatively short (greater than 100 points) and noisy time-series data (as in this case, because IMU recordings can be noisy). Distribution entropy (DistEn) is also a recent entropy measure, which has been developed based upon the probability density of vector-to-vector distances in state space. Further details on Distribution Entropy are presented in Reference [30].

It is not known a priori which features are more relevant for classification and a feature selection process is adopted. An additional reason to reduce the number of features used for classification is related to the curse of dimensionality in machine learning. Because a small dataset (20 paths for 12 vehicles) is used in this analysis, a limited number of features should be used for classification to avoid the curse of the dimensionality [31]. In this paper, we adopt the rule of thumb described in Reference [32] which states that there should be at least 5 samples for dimension. In this case, we adopt a number of features in the range of 4 to 6 (20 samples for each car divided by 5 is equal to 4). Experimental evaluation also shows that the use of all the features decreases the accuracy (e.g., 0.818 using all features against 0.832 with the 4 best features with sample rate of 250 Hz and $Gyro_{y}$). Because of its simplicity and time efficiency, in this paper, we adopt the RelieFF algorithm, which is part of the family of wrapper feature selection algorithms. The RelieFF algorithm [33] is based on the estimate of the quality of attributes according to how well their values distinguish between instances that are near to each other. The estimate can be implemented using the K-Nearest Neighbors (KNN) algorithm. Further details are presented in Reference [33].

#### 4.6.2. Spectral Domain Approach

**Figure 8:**
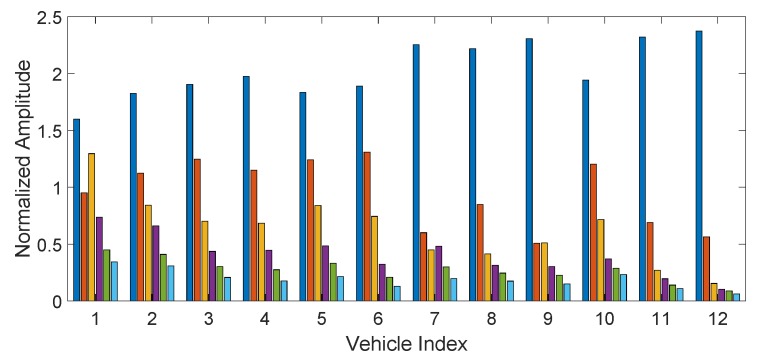
Amplitude of the components ($N_{freq}=6)$ in the frequency representation for each of the 12 vehicles using Gyroscope Y and the 500 Hz sample rate.

The other technique is based on the transformation of the signal from the IMU recording in the frequency domain using the FFT. Then, the frequency domain representation is divided in segments (i.e., frequency bands) with a number $N_{freq}$, which is one of the hyper-parameters identified in the Section 5. The Root Mean Square (RMS) of each frequency band is calculated to obtain $N_{freq}$ features. This approach is similar to the continuous authentication approaches for human beings, which are described in the related work in References [13,17]. The rational for this approach in vehicle authentication is that the main components of a vehicle (e.g., tyres, absorbers and wheels) will have smoother or harder reactions to road irregularities depending on the vehicle model. The smoother or harder response corresponds respectively to a lower occupation of the frequency bands or a higher occupation of the frequency bands. This can be seen from Figure 8, which shows the amplitude frequency representation with $N_{freq}=6$ of each vehicle for the same segment I using a sample rate of 500 Hz and $Gyro_{y}$. It can be seen from the Figure that the first 6 vehicles (all Pandas) have a similar distribution of the amplitude coefficients, while the other vehicles have a different distribution. In more detail, the amplitude of the second frequency component is only slighter less than the first frequency component, while there is wide gap between the amplitude of the first and second frequency components in the vehicle number 12, which is of a completely different brand and model and more importantly of a different class—sport car. On the other side, the frequency response can depend by many different factors including the speed of the vehicle and how it can change from one lap to another. Then, the goal is to identify the optimal hyper-parameters, which can optimize the vehicle classification regardless of the vehicle speed. Note that the frequency representation of a time series is complex and it is not known a priori which components of the time series representation are useful for classification. As shown in the results Section 5, the magnitude component of the frequency domain representation is much more relevant for classification than the phase component. Because of these reasons, the amplitude component of the FFT transform is used. It could be argued that other spectral representations apart from the one based on FFT could also provide a good classification accuracy. This paper has also used a wavelet based representation but the results are slightly worse than the frequency domain representation as shown in Section 5.

### 4.7. Machine Learning

The authors of this paper used different machine learning algorithms to produce the results shown in Section 5. Considering that it is a small subset of data, only shallow machine learning algorithms have been used. A brief description of the algorithms is provided here.

- K-Nearest Neighbors (KNN) classifies a data sample (called a query point) based on the labels of the near data samples. Different functions can be used to determine how near or distant are the nodes. The most common function is the euclidean distance but other distance metrics can be used like the Mahalanobis or Minkowski distance. The advantage of the KNN is that it is computationally efficient and it does not need high computational power in the training phase, while the classification phase could be more computational intensive than other algorithms. Apart from the distance metric, the K parameter must also be optimized.
- Decision Trees, where the algorithm iterates through the input data by using the features properties to reach a specific category, which is more similar to the labeled data. The implementation of decision trees is usually very simple and fast if the data is well structured. An additional advantage is that they performs well even with high dimensional datasets. The disadvantages are the long training time and that the orders of the features in tree nodes have adverse effect on performance. In this paper, the maximum number of splits is the hyper-parameter, which must be optimized. Because individual decision trees tender to overfit, ensemble methods are also used. In particular, we have used the Random Forest algorithm (bagged decision trees) and the Ada Boost algorithm as well. The Ada Boost algorithm has been used in combination with the Decision Tree.
- SVM is a supervised algorithm, which learns to classify the data points (e.g., originating from the observables), from the labeled training samples (e.g., the reference fingerprints). SVM separates the labeled set in two areas on a multi-dimensional surface by using a separating function, which can be of different types—linear, Radial Basis Function (RBF), polynomial, sigmoidal are the most common. Because the multi-dimensional surface is divided in two areas, SVM is a binary classifier and it can be directly used to distinguish between two mobile phones or for validation (to validate that the claimed identity of a mobile phone). The extension of SVM to multi-classifier identification has been proposed by various authors. In this paper the OneVsOne approach is used. The RBF kernel is used and the RBF scaling factor γ must be optimized together with the C factor of the SVM algorithm.

Two main classification metrics are used—(a) the confusion matrices where each row of the matrix represents the instances in a predicted class while each column represents the instances in an actual class and (b) the identification accuracy, which is the sum of the true positive plus the true negatives divided for the total number of samples.

## 5. Results

### 5.1. Hyper-Parameters to Optimize

This section describes the results of the methodology proposed in the previous sections using the two main different techniques—the first technique based on the use of statistical features and the second technique based on the frequency domain (i.e., spectral approach). Both techniques were applied to the segments of the path driven by the vehicles. Various hyper-parameters are present in the analysis and they are further described here:

- the specific segment of the path driven by the vehicle. Each segment described in Figure 1 has its own characteristics and it is interesting to evaluate how the characteristics of each segment may impact the vehicle classification. The analysis is conducted on the seven segments identified in Figure 1 and Figure 7.
- the IMU component and the direction element (X,Y,Z). An initial selection is based on the consideration that only directions which are directly related to the surface of the road can be used for vehicle authentication as the other directions are biased by the latitudinal and longitudinal movements of the vehicle (e.g., when turning a bend). Then, the optimal directions would be Accelerometer in the *Z* direction (i.e., the vertical direction) and the Gyroscope in the *X* and *Y* directions (the horizontal directions).
- the sample rate of the IMU recordings on which the feature extraction or the spectral domain is applied.
- another relevant choice in the features-based approach is the selection of the statistical features. Because it is a small dataset (20 paths for 12 vehicles), a limited number of features should be used for classification to avoid the curse of the dimensionality [31]. In this paper, a wrapper feature selection approach is used based on the RelieFF algorithm [33] to select the optimal subset of features.
- in the spectral (i.e., frequency domain) based approach, there is also the need to select a limited number of features (i.e., the spectral bands). In this case, the selection is on the number of bands. As shown in the subsequent sections, the RelieFF approach is also used for a large number of frequency bands.
- the hyper-parameters used in the machine learning algorithms used to perform the classification must also be optimized (e.g., K in the KNN algorithm).

The comparison of the hyper-parameters will be described for each technique—statistical features Section 5.2 and spectral domain Section 5.3.

### 5.2. Statistical Features Approach

Here we provide the results for the application of the statistical features approach. As mentioned before, one of the hyper-parameters (common to both to the features approach and the spectral approach) is the choice of the component and the direction.

**Figure 9:**
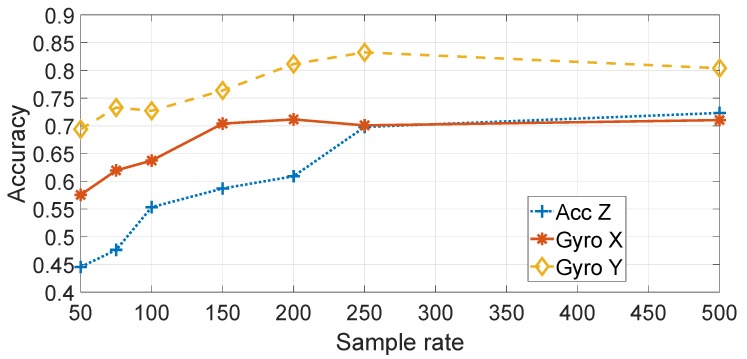
Comparison of different elements and directions of the IMU with feature based approach (sample rate in Hz).

Figure 9 shows a comparison of the different components (e.g., accelerometers and gyroscopes) and directions (e.g., Z,Y) for the application of the statistical features approach on the basis of different sample rates. The SVM machine learning algorithm was used. As discussed before, only the Accelerometer in the *Z* direction ($Acc_{z}$ in the rest of this paper), the Gyroscope in the *X* direction ($Gyro_{x}$ in the rest of this paper) and the Gyroscope in the *Y* direction ($Gyro_{y}$ in the rest of this paper) are considered. As discussed before, the direction has been adjusted from the initial recordings to be in the direction of travel of the vehicle. Figure 9 shows that the highest classification accuracy is obtained by using $Gyro_{y}$, which is reasonable, because the pitch of the vehicle is more strongly stimulated by irregularities in the road surface. Figure 9 also shows that the accuracy improves with the increase of the sample rate. This result may be explained by the consideration that a higher sample rate provides more details and more discriminating power in the application of the features or the spectral transform with the machine learning classification. Evidence from studies on device identification in general supports this hypothesis [26]. As the classification is higher with $Gyro_{y}$, the subsequent results are based on $Gyro_{y}$.

In the rest of this section, it is discussed more in detail how the statistical features were selected to produce the results of Figure 9.

As discussed in the methodology Section 4, the approach based on the statistical features uses the 10 features described in Section 3, but a subset of features is selected using the RelieFF algorithm, where the best four features are selected. The number four was chosen because it was found out that other features do not contribute significantly on the basis of the weight ranking and to avoid the curse of dimensionality because of the small sampling set.

**Figure 10:**
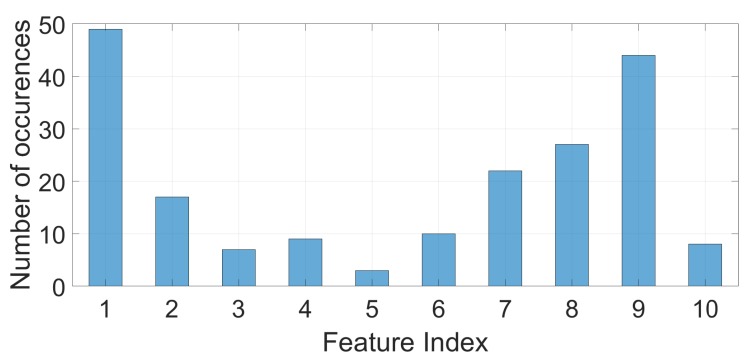
Histogram of the features across all the segments and sample rates.

The histogram in Figure 10 shows the occurrences of the best four ranked features of RelieFF across all the best seven selected segments and the different samples rates (i.e., for each segment and each sample rate the highest four ranking features are selected) for $Gyro_{y}$. The histogram shows that the most relevant features are—feature 1 (Variance), feature 8 (Permutation Entropy with M=4) and feature 9 (Approximate Entropy).

**Figure 11:**
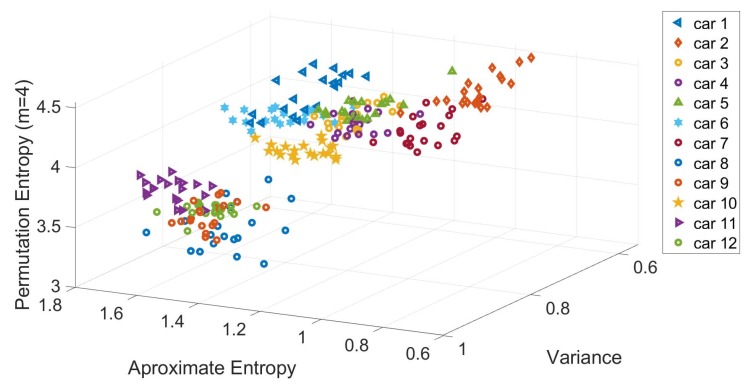
Scatter plot based on the three main features derived from the RelieFF algorithm. Gyroscope Y direction at 500 Hz and Segment I.

A visual representation on how the features identified from Figure 10 are relevant for the classification of the specific vehicles is shown in Figure 11, where a scatter plot for three selected features mentioned above (i.e., Variance, Permutation Entropy and Approximate Entropy) is shown. The scatter plot is based on Gyroscope Y with a sample rate of 500 Hz. Each of the points shown in the scatter plot of Figure 11 represents each of the EL for each vehicle.

**Figure 12:**
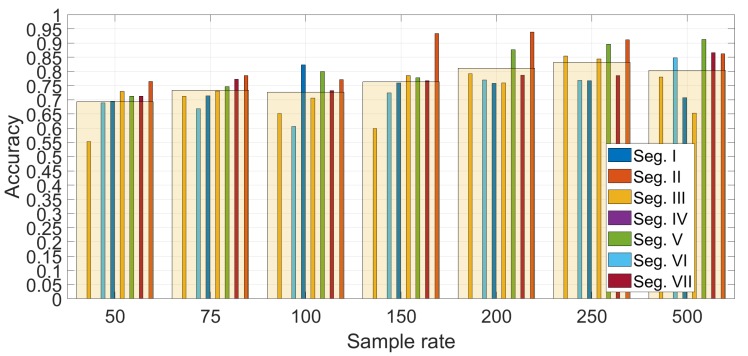
Comparison of the different segments and different sample rates (sample rate in Hz) for the features approach.

Figure 12 shows the detail on the accuracy obtained foreach segment using $Gyro_{y}$ and SVM with optimized hyper-parameters ($\gamma =2^{3}$ and $C=2^{7}$). The average values across segments are also indicated with a transparent bar for each sample rate. The Figure 12 provides two significant results—the first is that the sample rate greatly impacts the classification accuracy. a higher sample rate provides more discriminative value than a lower sample rate as the identification accuracy increase steadily with the sample rate from 50 Hz to sample rate equal to 250 Hz. Then, an even higher sample rate does not improve the identification accuracy. This is an important result, because it shows that there is no need to use very high sample rates, which would not be practical for a deployment of continuous authentication using smartphones (i.e., current mass market smartphones have a sample rate around 100 Hz–200 Hz). The second result is that the choice of the segment can impact the identification accuracy. The Figure shows that the identification accuracy can vary greatly among the different segments and these results for the statistical features approach (a similar analysis is provided below for the spectral approach) can provide an insight into which road segments can be preferred in this authentication approach. For example, it can be seen that the segment III provides across all the sample rates a lower identification accuracy than the other segments. If we compare the results in Figure 12 with the results in Figure 7, it can be seen that segment II provides in general a higher identification accuracy (especially with sample rates at 150 Hz and 200 Hz) which can be related to a higher variance of the accelerometer in the Z direction due to the presence of irregularities of the road surface including the speed bump SB03. Then, we can conclude that the presence of irregularities on the road surface or a greater road roughness can provide a higher identification accuracy. This also means that smooth surfaces like a highway may provide a lower identification accuracy. Segment III, which does not provide a high classification accuracy, does not have specific road surface irregularities (see the map in Figure 1) and it could be considered similar to the Highway case.

The results, shown in the previous figures, confirm that it is possible to obtain a significant classification accuracy—higher than 80% in the case of a sample rate of 200, 250 and 500 Hz for SVM and $Gyro_{y}$. On the other side, it will be demonstrated in the subsequent sections that it is possible to obtain an even higher classification accuracy using the spectral approach whose results are presented in Section 5.3.

**Figure 13:**
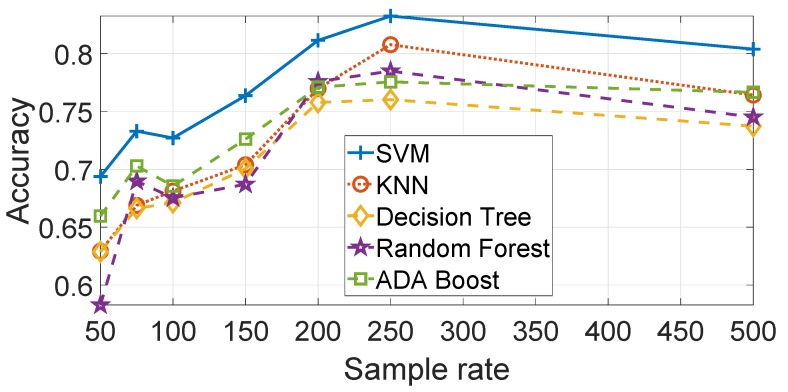
Comparison of machine learning algorithms with feature based approach (sample rate in Hz). Gyroscope Y direction. Results are based on the average across all segments.

As discussed in the Section 4, different machine learning algorithms are used to produce the results. A comparison of the results using the $Gyro_{y}$ data are provided in Figure 13. Each of the machine learning algorithms have been optimized in relation to their hyper-parameters. The optimized ML parameters are—SVM with $\gamma =2^{3}$ and $C=2^{7}$, KNN with Euclidean Distance and K=1 and Decision Trees with maximum number of splits =3. The Random Forest and Ada Boost algorithms (based on the Decision Tree) have also been optimized using the auto-optimization function of Matlab. Figure 13 shows that SVM has a better performance accuracy than the other machine learning algorithms, in particular KNN and the Decision Tree. Random Forest and Ada Boost performs better than the Decision Tree but less than SVM. The result is consistent for all the sample rates. Similar results are obtained for $Acc_{z}$ and $Gyro_{x}$ but they are not presented to avoid an excessive length of this paper.

As the identification accuracy may not provide a comprehensive view of the false positive or false negative, confusion matrices are provided for $Gyro_{y}$ at different sample rates. In all confusion matrices, the SVM algorithm was used.

**Figure 14:**
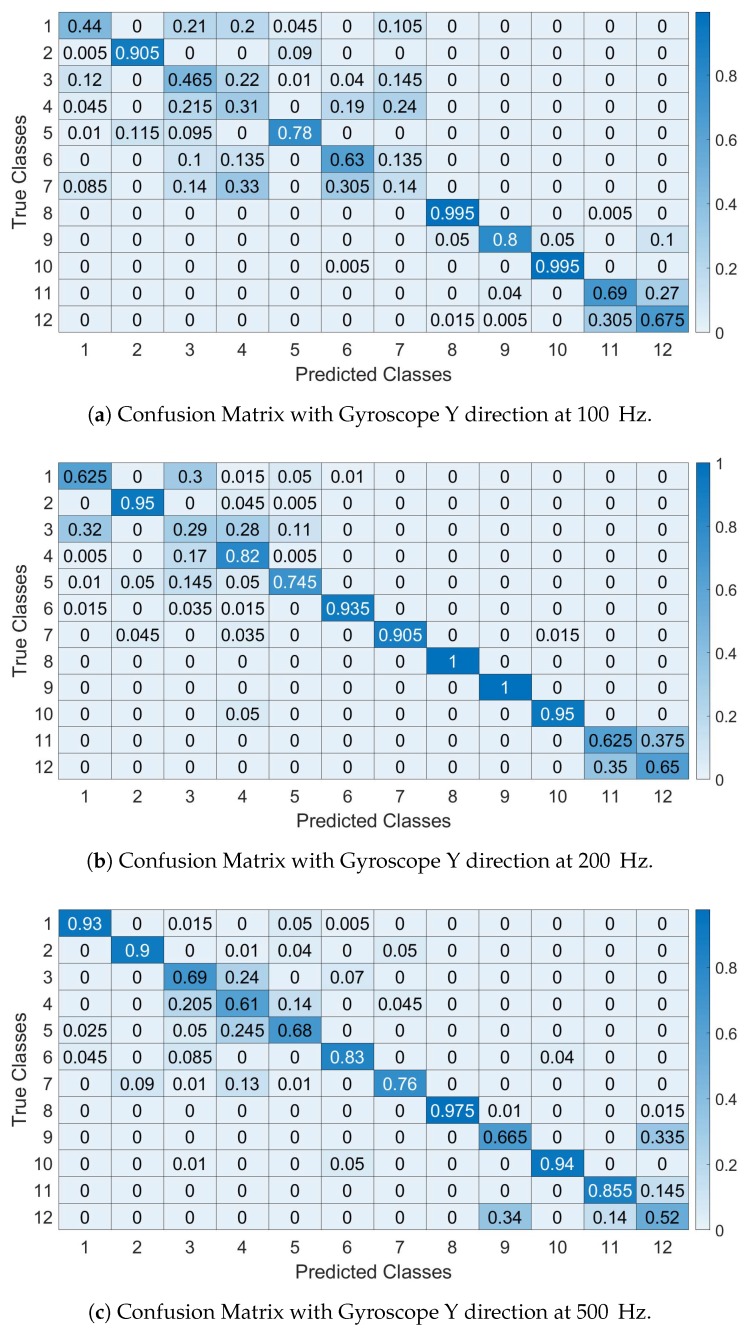
Confusion Matrices with Features approach. The Y axis represents the True classes and the X axis represents the Predicted classes (**a**–**c**).

The confusions matrices are shown in Figure 14a–c (respectively at 100, 200 and 500 Hz) for $Gyro_{y}$ and they are calculated for Segment I (similar results are obtained for the other segments) for the sample rate and IMU components indicated in the figure. They confirm the previous results on the identification accuracy where smaller sample rates decrease the identification accuracy and provide more insights on how the specific vehicles are classified. In particular, the confusion matrices show that the first 6 vehicles (e.g., of the same model Fiat Panda) are more difficult to classify with high accuracy as the number of False Positive and False Negatives is relevant. It is also noted that the 7th car (e.g., Fiat Punto) is of the same brand and it shares similar features of the Panda (e.g., age and vehicle class).

**Figure 15:**
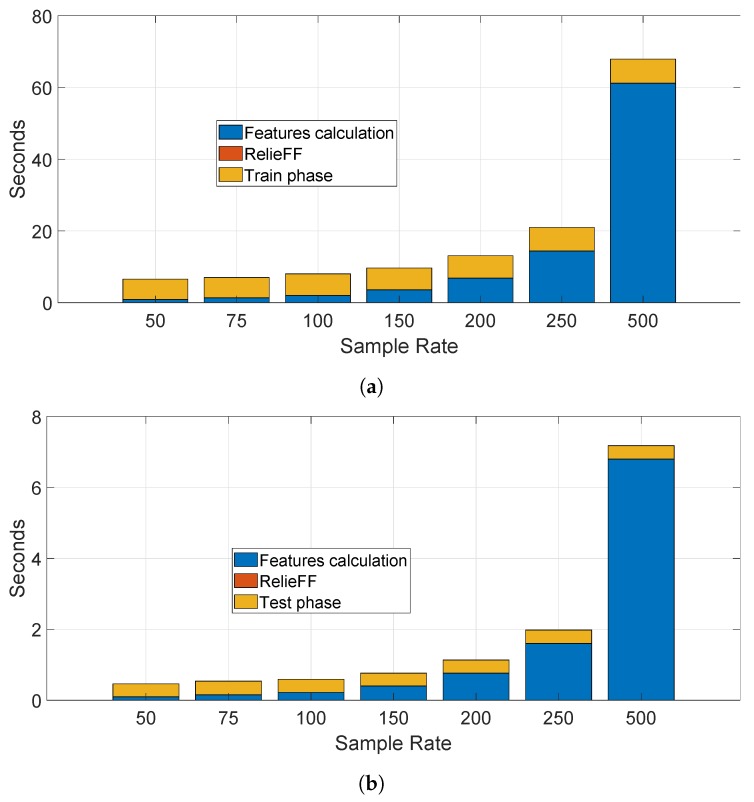
Time needed to execute the statistical features approach (sample rate in Hz). (**a**) Time needed to execute the training phase using the statistical features approach and the Ada Boost algorithm; (**b**) Time needed to execute the testing phase using the statistical features approach and the Ada Boost algorithm.

We have also calculated the time needed to process the data, perform the training and testing for a specific segment (i.e., segment II) for the statistical features approach. This evaluation is useful for a practical application of this technique. We present the result for the Ada Boost algorithm (based on the Decision Tree) as this algorithm had the longest computational time among all the algorithms. The results are presented in the bar stacked in Figure 15a for the training phase and in the bar stacked in Figure 15b for the testing phase for different sample rates. We note that the calculation of the statistical features require the larger portion of the processing time, while the selection feature with RelieFF is negligible (it can be detected with difficulty in the figures). Obviously, the computational time increases with the sample rate as a greater sample rate means a higher number of sample to be processed by the feature extraction process. The time needed to identify and authenticate a car is only few seconds on the computing platform used to perform the computation—a laptop with Intel I7-8550U with a clock at 1.8 GHz with 16 GB of RAM. A more powerful computing platform would be able to considerably reduce this processing time. Taking in consideration that the optimal sample rate for the accuracy was 250 Hz, the identification time would be approximately 2 s in the optimal case. Similar considerations are repeated for the spectral approach in the next section with a much shorter time-frame because of absence of a feature extraction process.

### 5.3. Spectral Approach

As described in the methodology Section 4 the spectral approach (i.e., in the frequency domain) is based on the application of the FFT to the IMU recordings. Then, the frequency representation is divided in an equal number of frequency bands and the total amplitude power is calculated for each band. The obtained values (i.e., the coefficients) are used for classification. As in the case of the features, the dimensionality reduction is significant: $S_{rate}->N_{freq}$ (e.g., from 200 Hz to 6 coefficients).

**Figure 16:**
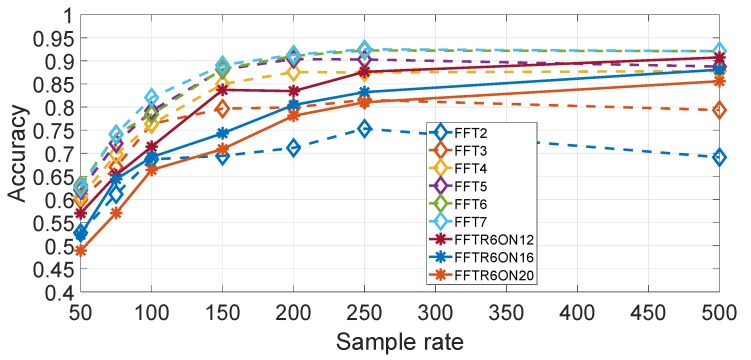
Comparison for different values of with spectral approach (sample rate in Hz). Gyroscope Y direction. Results are based on the average across all segments.

In this approach, the hyper-parameter is the number of coefficients in the spectral domain. Then, the optimal value of $N_{freq}$ must be identified but $N_{freq}$ cannot be large because of the curse of the dimensionality, then it is decided that $N_{freq}$ is limited to 6. On the other side, a feature selection process as in the feature approaches can be used with a value of $N_{freq}$ greater than 6. Figure 16 shows a comparison of different values of $N_{freq}$ using $Gyro_{y}$ for different values of the sample rates, where only a limited set is used when $N_{freq}$ is greater than 6. It can be seen that the optimal performance curve is obtained for $N_{freq}$. We note that a value of $N_{freq}=7$ was also used to make a comparison (thus contrary to the rule defined above), but it can be seen that there is no significant improvement of $N_{freq}=7$ against $N_{freq}=6$. The use of higher numbers than $N_{freq}=7$ ($N_{freq}=12$, $N_{freq}=16$ and $N_{freq}=20$) in combination with RelieFF shows that the identification accuracy is worst than with $N_{freq}=6$. By comparison, the results obtained in the previous section for the feature approach, it can be concluded that a higher accuracy is obtained by using the spectral approach than the feature approach. Two potential explanations are possible. One explanation is related to the physical response of the vehicle against irregularities in the road as different vehicles types have different automotive suspension components (e.g., coil springs and control arms), which produce a different frequency response on the same road segment. This can be a more distinguishing factor for vehicle types than the designed statistical features used in the feature approach. The second aspect is that the selected features in the feature approach are derived from the research literature on continuous authentication of human beings, which may not be fully appropriate to the continuous authentication of vehicles.

**Figure 17:**
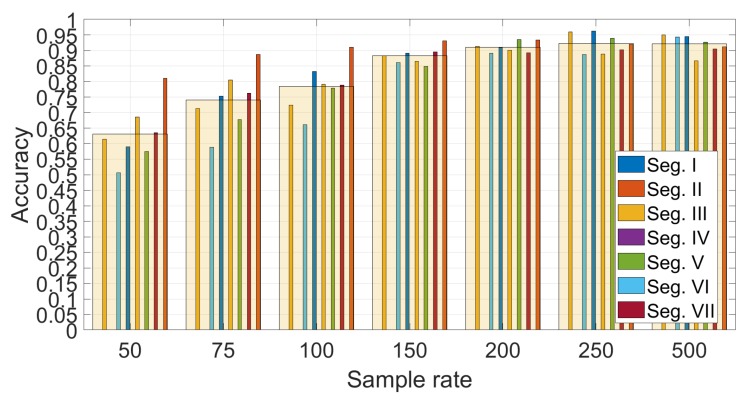
Comparison of the different segments and different sample rates for the spectral approach (sample rate in Hz).

The results are obtained using the $Gyro_{y}$ direction and by averaging the results across the 7 segments selected in the study. A more detailed study for the different segments is shown is provided in Figure 17 for $N_{freq}=6$, which confirms the previous results.

**Figure 18:**
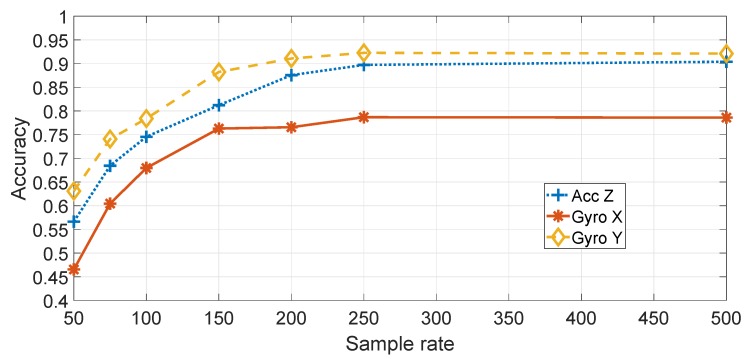
Comparison of different elements and directions of the IMU with spectral approach (sample rate in Hz). Results are based on the average across all segments.

In a similar way to the features approach, a comparison was made among the main IMU components and directions and the results are presented in Figure 18, which confirms the result of the features approach, as the best identification accuracy is obtained with $Gyro_{y}$. The results are obtained by averaging the results across all segments, using SVM withe optimized values of the machine learning hyper-parameters.

**Figure 19:**
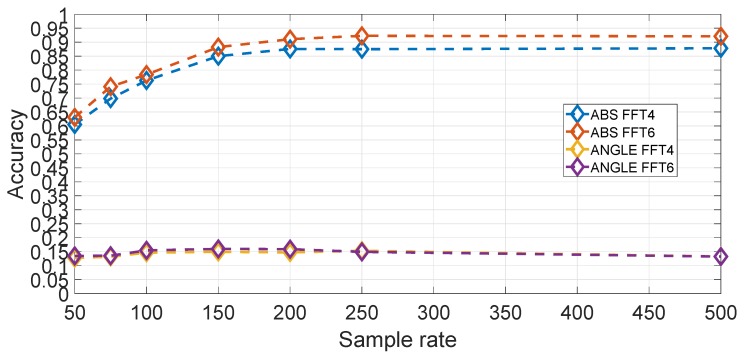
Comparison of magnitude and phase components in the spectral approach using $N_{freq}=6$ for the Gyroscope Y direction (sample rate in Hz). Results are based on the average across all segments.

In comparison to the feature based approach, another hyper-parameter to select is the amplitude or phase component as the FFT provides complex values. An evaluation using $Gyro_{y}$, $N_{freq}=6$ and averaged on all segments for different sample rates is shown in Figure 19. It can be seen that the amplitude component is much more significant than the phase component. The reason is that the amplitude part is directly related to the frequency response of the mechanical components (i.e., coil springs and wheels) of the vehicle while the phase is not (e.g., the reaction of vehicle to a bumper is mostly related to the speed of the vertical acceleration).

**Figure 20:**
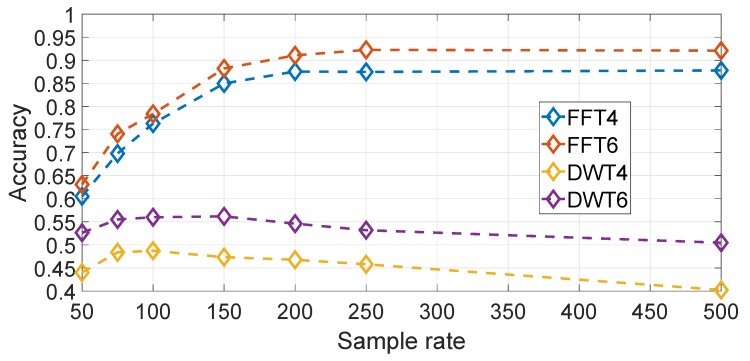
Comparison of spectral and wavelet approaches (sample rate in Hz). Gyroscope Y direction at 500 Hz. Results are based on the average across all segments.

It could also be imagined that other transforms rather than the FFT transform could give better classification results. This hypothesis is evaluated in Figure 20 where a wavelet based transform (i.e., based on a Daubechies wavelet of order 10) is compared against the frequency domain approach for different sample rates, $Gyro_{y}$ and $N_{freq}=6$ both for wavelet and frequency domains representations. In both cases, the amplitude component of both transforms have been used. The result in Figure 20 shows clearly that the FFT based approach is significantly better than the wavelet based approach. Different wavelets have also been used with similar results.

**Figure 21:**
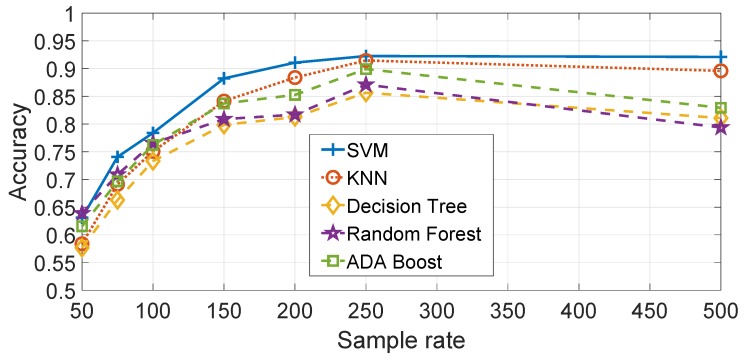
Comparison of machine learning algorithms with spectral based approach (sample rate in Hz). Gyroscope Y direction at 500 Hz and $N_{freq}=6$. Results are based on the average across all segments.

All the previous results are obtained by using the SVM machine learning algorithm. As in the feature based approach, a comparison is performed among the SVM, KNN and Decision Tree algorithms and the results are shown in Figure 21. The results are created by averaging the classification accuracy results for all the segments. It can be seen that SVM outperforms KNN, Decision Tree and the related ensemble methods like Random Forest and AdaBoost. The optimized ML parameters are: SVM with $\gamma =2^{4}$ and $C=2^{8}$, KNN with Euclidean Distance and K=1 and Decision Trees with maximum number of splits = 5. The Random Forest and Ada Boost algorithms (based on the Decision Tree) have also been optimized using the auto-optimization function of Matlab.

As in the case of the feature based approach, confusion matrices are presented in the rest of this section. The confusion matrices are calculated by averaging the results across all the segments for the sample rate and IMU component indicated in the respective figure.

**Figure 22:**
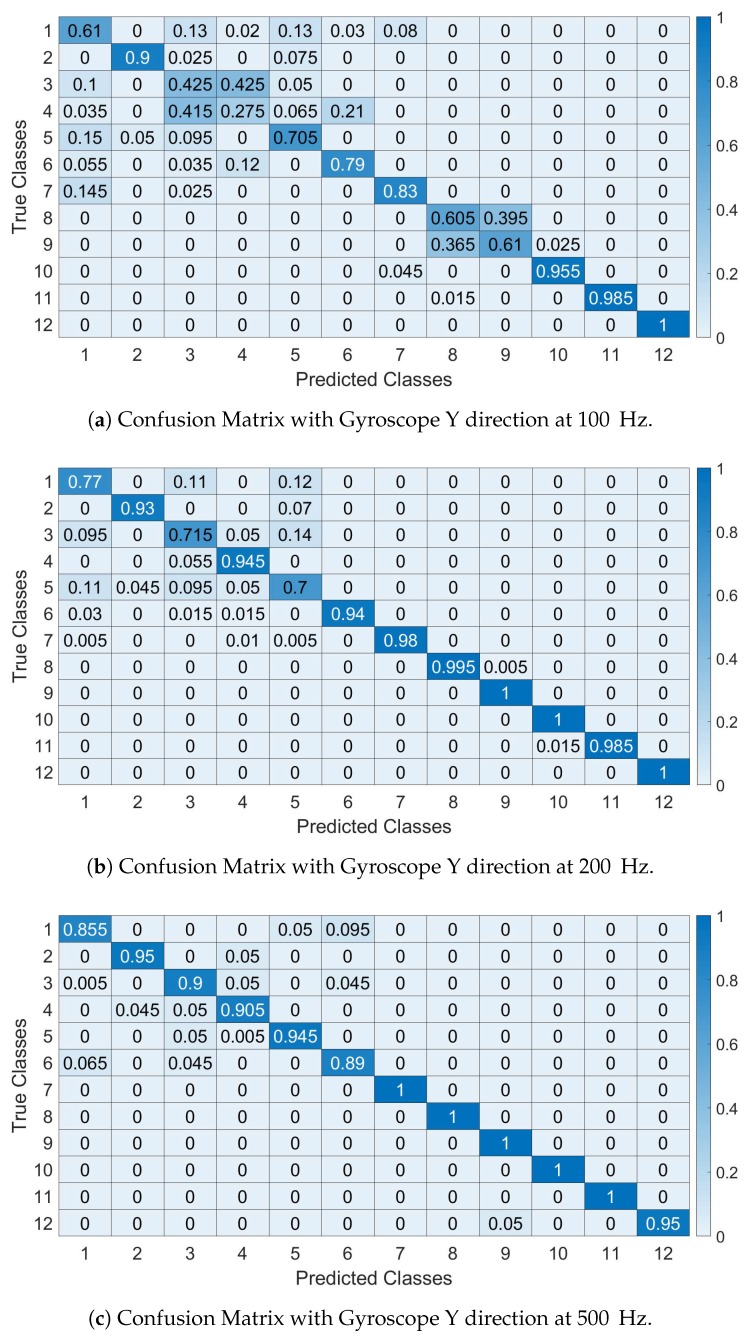
Confusion Matrices with Spectral approach. The Y axis represents the True classes and the X axis represents the Predicted classes.

Figure 22a–c show the confusion matrices using the spectral approach with Gyroscope Y for Segment I (similar results are obtained for the other segments) and with an IMU recording sampled respectively at 100 Hz, 200 Hz and 500 Hz. The confusion matrices confirm the previous Figures obtained for the identification accuracy. As in the case of the feature based approach, the first 6 vehicles (i.e., of the same Panda model) are more difficult to distinguish than the other vehicles as expected because they have similar mechanical features.

**Figure 23:**
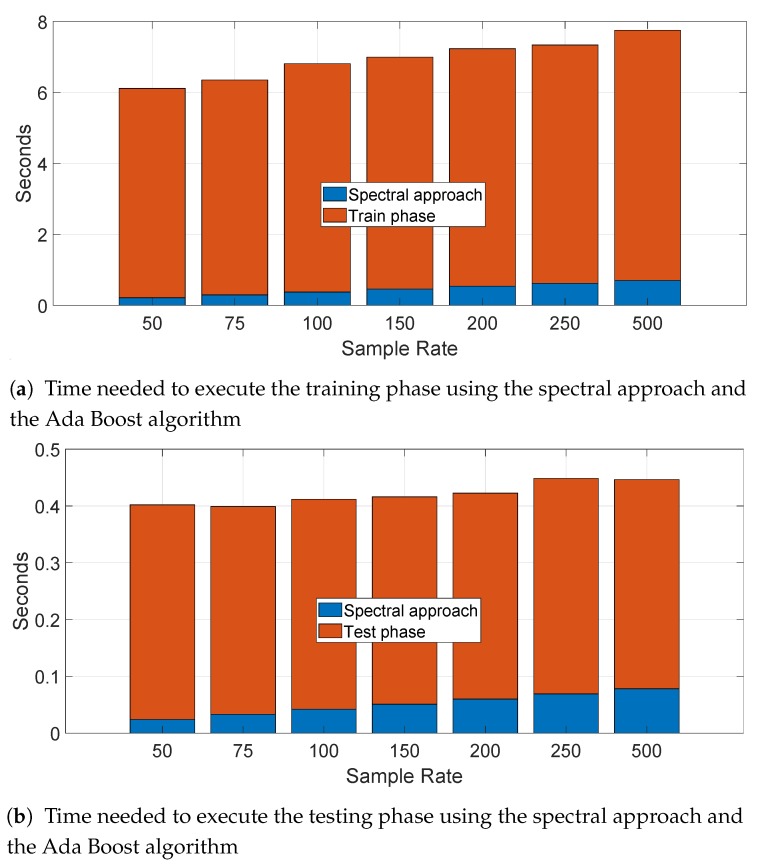
Time needed to execute the spectral approach (sample rate in Hz). (**a**) Time needed to execute the training phase using the spectral approach and the Ada Boost algorithm. (**b**) Time needed to execute the testing phase using the spectral approach and the Ada Boost algorithm.

As in the previous case of the statistical feature approach, we have also calculated the time needed to process the data, perform the training and testing for a specific segment (i.e., segment II) for the spectral approach. We present the result for the Ada Boost algorithm (based on the Decision Tree) as this algorithm had the longest computational time among all the algorithms. The results are presented in the bar stacked Figure 23a for the training phase and in bar stacked Figure 23b for the testing phase for different sample rates. In comparison to the statistical feature approach, the processing time is much lower and it is possible to perform an identification and authentication in less than a second (0.4 s for sample rate at 250 Hz).

## 6. Discussion and Conclusions

The results presented in the previous section confirmed that the continuous authentication approach already proposed for human beings on the basis of their gait and IMU data can also be applied to vehicles as the spectral approach provides an identification accuracy higher than 90% and even higher than 95% for some specific road segments. The results are obtained using extensive measurements campaigns with 12 different vehicles on tens of kilometers of road. Even if the data were collected at a high sample rate using professional IMU, the analysis was performed with lower sample rates, which are obtainable with modern mobile phones (e.g., 200 Hz). The same driver was used to collect the recording in all the vehicles to avoid the introduction of the bias of the driver. We highlight that the proposed approach for continuous authentication of vehicles was derived from the approaches (e.g., statistical features or spectral approaches) already defined in the literature for continuous authentication of human beings. A comprehensive analysis and optimization of the hyper-parameters were performed on the dataset to select the optimal values, approaches and algorithms. Such optimization can be useful for future research activities by the research community. The results show that the spectral approach provides a higher identification accuracy than the feature approach and we recommend its use. Future developments may take in consideration more sophisticated time-frequency representation even if the amount of data to be analyzed will be higher. The presented approach is based on a strong dimensionality reduction from the initial IMU recording, which make it suitable for practical applications as only a small subset of data (e.g., 6 amplitude values in the spectral domain) for a road segment of hundreds of meters must be sent to the remote authentication system.

A complementary point of view is that the identification of vehicles can also generate a privacy threat. Then, a complementary research activity would be to investigate techniques to remove the discriminating features used to identify the vehicle, but still preserve the information needed for traffic applications (e.g., road surface maintenance, traffic analysis).

Future developments will investigate the combination of data from different components and directions to obtain a higher identification accuracy. The application of de-noising techniques to improve the quality of the recorded data could also be used to improve the classification accuracy but it must be ensured that the application of de-noising algorithms does not remove the discriminating characteristics of the vehicle. Finally, the entire dataset is made available to the research community to perform additional studies in this area.

Future extensions of this paper will also investigate the impact of different variables on the identification and authentication accuracy. For example, if the same vehicle has different numbers of passengers or if the driver is different as the driving style can impact the continuous authentication approach.

## Figures and Tables

**Table 1:** Description and GNSS position of points recognised in the EL.

Name	Description	GPS Lat.	GPS Lon.
SB01	sequence of ten rumble strips in the right driving lane	45.811832	8.627199
SB02	speed bump before the roundabout OP02	45.811845	8.627569
SB03	speed bump after the roundabout OP03	45.811883	8.628583
SB04	speed bump in front of the Visitor’s center	45.808842	8.632676
RF06	small road fix only in the center of the road	45.811370	8.632282
RF15	long road patch through both driving lanes	45.809502	8.628149

**Table 2:** Order and specifications of used cars.

Car	Manufacturer	Model	Gen.	Version
1	Fiat Automobiles	Panda	2nd	Active
2	Fiat Automobiles	Panda	2nd	Active
3	Fiat Automobiles	Panda	2nd	Active
4	Fiat Automobiles	Panda	2nd	Active
5	Fiat Automobiles	Panda	2nd	Active
6	Fiat Automobiles	Panda	2nd	Active
7	Fiat Automobiles	Punto	2nd	3-door
8	Fiat Automobiles	Doblo	1st	Facelift
9	Fiat Automobiles	Tipo	3rd	Hatchback
10	Mitsubishi	Colt	6th	CZ3
11	Škoda Auto	Octavia	3rd	Estate
12	Mazda Motor Corp.	Mazda3	4th	Hatchback

**Table 3:** Statistical Features used for dimensionality reduction.

Feature Id	Feature Description
1	Variance
2	Skewness
3	Kurtosis
4	Shannon Entropy
5	Wavelet Spectral Shannon Entropy
6	Wavelet Spectral Log Entropy
7	Permutation Entropy with Embedding dimension 3
8	Permutation Entropy with Embedding dimension 4
9	Aproximate Entropy
10	Distribution Entropy

## Abbreviations

The following abbreviations are used in this manuscript:

ApEn	Approximate entropy
BNW	Background Noise Window
DistEn	Distribution entropy
DTW	Dynamic Time Warping
EL	Experimental Lap
FFT	Fast Fourier Transform
GNSS	Global Navigation Satellite Systems
IMU	Inertial Measurement Unit
ITS	Intelligent Transport Systems
JRC	Joint Research Centre
KNN	K-Nearest Neighbors
OP	Orientation Point
PE	Permutation Entropy
PIN	Personal Identification Number
PKI	Public Key Infrastructure
RBF	Radial Basis Function
RF	Road Feature
RMS	Root Mean Square
SB	Speed Bump
SVM	Support Vector Machine
